# Vemurafenib, cetuximab and camrelizumab in BRAF V600E-mutated/MSS metastatic colorectal cancer

**DOI:** 10.1186/s12967-025-07312-6

**Published:** 2025-11-12

**Authors:** Gui-Xia Wei, Yu-Wen Zhou, Peng Cao, Wei-Bing Leng, Li Wang, Jie Tang, Meng Qiu

**Affiliations:** 1 Department of Colorectal Cancer Center, West China Hospital of Sichuan University, 37 Guoxue Xiang Street, Chengdu, Sichuan Province 610041 China; 2 Clinical Trial Center, West China Hospital, Sichuan University, Chengdu, Sichuan China

**Keywords:** BRAF V600E mutation, Metastatic colorectal cancer, Vemurafenib, Cetuximab, PD-1 monoclonal antibody, Phase I study

## Abstract

**Introduction:**

Patients with BRAF V600E-mutated/microsatellite stable (MSS) metastatic colorectal cancer (mCRC) are associated with a poor prognosis. Backline treatment has minimal efficacy. Multi-target inhibitors of the RAS-RAF-MEK signaling pathway combined with PD-1 monoclonal antibody may be a promising strategy for BRAF V600E-mutated mCRC.

**Methods:**

This prospective phase I trial enrolled patients to explore tolerability and safety of the VCC regimen in previously treated patients with BRAF V600E-mutated/MSS mCRC. Enrolled patients treated with VCC therapy every 2 weeks (cetuximab 500mg/m^2^; camrelizumab 200 mg; vemurafenib 960 mg orally once daily). Adverse events and efficacy were monitored and recorded throughout the administration and follow-up period.

**Results:**

This trial enrolled 12 eligible patients. Total 2 patients had DLT: one had grade 3 thrombocytopenia and immune myocarditis, another had grade 3 vaginal bleeding. Adverse events (AEs) of grade 3 or higher occurred in 50% of patients. Grade 3 AEs were mainly drug-related fever (25.0%), drug-related rash (16.7%). Median overall survival (OS) and PFS were 7.19 months and 3.47 months, respectively. The ORR and DCR were 33.3% and 66.7%. One patient achieved CR. three patients achieved PR. An abnormal CD4/CD8 ratio was associated with a higher risk of progression. Different efficacy of VCC regimen may be attributed to the difference in tumor immune environment.

**Conclusions:**

A combination of vemurafenib, cetuximab combined with camrelizumab exhibited manageable adverse reactions and efficacy in BRAF V600E-mutated/MSS patients with metastatic colorectal cancer who progressed after standard treatment. This is a pilot study and a larger phase II trials is planned to validate the findings. (ClinicalTrials.gov ID: NCT05019534).

**Supplementary Information:**

The online version contains supplementary material available at 10.1186/s12967-025-07312-6.

## Introduction

BRAF mutation is present in about 5–15% of metastatic colorectal cancer (mCRC). And the BRAF V600E mutation accounts for approximately 95% of BRAF-mutated colorectal cancers [1, 2]. The prognosis of mCRC with BRAF V600E mutation is poor, with median overall survival (OS) from the first line therapy of approximately 10–17 months [3]. At present, doublet or triplet chemotherapy (based on fluorouracil, combined with platinum or (and) irinotecan) plus bevacizumab is currently the recommended first-line treatment for BRAF V600E-mutated mCRC [4–6].

After failure of initial therapy, subsequent lines of treatment are associated with minimal effect, and rapidly progressive disease is observed. Although BRAF is a driver mutation in several tumor types, and BRAF inhibitors have clinical activity in BRAF V600E–mutated non–small cell lung cancer (NSCLC) and melanoma, previous studies have shown that anti-BRAF inhibitor monotherapy has a response rate of only 5% in previously treated BRAF V600E-mutated CRC [2, 7, 8]. Multi-targeted inhibition of the RAS-RAF -MEK pathway may be a potentially effective strategy for BRAF V600E-mutant mCRC by blocking the feedback reactivation of MAPK pathway signaling. Currently, the efficacy of the combination regimen of anti-BRAF inhibitor and anti-EGFR inhibitor has been demonstrated in several studies. The BEACON study also found that the triplet (encorafenib + cetuximab + binimetinib) and doublet (encorafenib + cetuximab) groups were associated with improved survival compared with the control group (cetuximab + irinotecan). In previously treated patients with BRAF V600E mCRC, the survival benefit of the triplet arm was not significantly higher than that of the doublet arm [9]. The SWOG S1406 study discovered that vemurafenib in combination with cetuximab and irinotecan significantly prolonged the progression-free survival (PFS) compared with cetuximab plus irinotecan in BRAF-mutant mCRC [10]. Since the BEACON data, encorafenib plus cetuximab is recognized as an effective treatment and is the current standard therapy after failure of at least one line of treatment, despite long-term outcomes remain poor.

In addition, there are multiple immune escape mechanisms in BRAF V600E mutated tumors. Recruitment of immunosuppressive factors (e.g., PD-L1, CTLA-4, and HLA-G) and immunosuppressive cells such as Treg cells have been detected in BRAF-mutated tumors [11, 12]. Moreover, the IMspire150 study demonstrated that the addition of PD-1 monoclonal antibody to targeted therapy was well tolerated and significantly improved PFS in advanced melanoma patients with BRAF V600E mutation [13]. Another study showed that combined PD-1, BRAF and MEK inhibition was associated with an ORR of 78% in advanced BRAF-mutant melanoma [14]. An increasing number of studies have shown that cetuximab has strong immunomodulatory activity, in part via antibody-dependent cell-mediated cytotoxicity (ADCC) effect, in addition to inhibition of the EGFR intracellular signaling pathway when combined with PD-1 monoclonal antibody in colorectal cancer [15–17]. The Fc region of cetuximab binds to the receptor found on NK cells (Fc receptor CD16/FcγRIII), resulting in NK cell via IFN-γ, chemokines, cytokines. Active NK cells can carry out their own lytic activity on tumor cells, and each active NK cell can serially lyse multiple target cells [15, 18]. NK cells facilitate crosstalk with dendritic cells (DCs) and other immune cells (e.g., macrophages, other NK cells) via IFN-γ and various cytokines. Activated NK cells that lyse tumor cells lead to the release of tumor antigens, which can be cross-presented by DCs to cytotoxic T cells, priming them for additional tumor cell killing activity [19–21].

Based on the above studies, BRAF inhibitor plus EGFR inhibitor combined with PD-1 monoclonal antibody may be a promising combination therapy strategy. Therefore, we conducted this prospective, phase I study to evaluate the safety and tolerability of the BRAF inhibitor (vemurafenib) plus EGFR inhibitor (cetuximab) and PD-1 monoclonal antibody (camrelizumab) (VCC) in previously treated patients with BRAF V600E-mutated/MSS type mCRC.

## Materials and methods

### Clinical trial design and oversight

This study is an open-label, single-center, phase I trial evaluating the safety and tolerability of the VCC regimen. Patients with previously treated BRAF V600E-mutated/MSS type mCRC will be assigned to receive the VCC regimen every 2 weeks.

The study protocol was approved by the Ethics Committee or Institutional Review Board and was conducted in accordance with the principles of the Declaration of Helsinki. Written informed consent was obtained from each patient.

### Patients

The key inclusion criteria including: Participants have histologically or cytologically confirmed diagnosis of adenocarcinoma of the colon or rectum, with clinical confirmation of unresectable and/or metastatic disease that is measurable according to Response Evaluation Criteria in Solid Tumors (RECIST 1.1); Prior treatment with at least one systemic treatment (chemotherapy or target therapy) for mCRC; Male or female ≥ 18 years of age; Presence of BRAF V600E mutation in tumor tissue and confirmation of MSS or pMMR status from NGS or IHC. Patients were not previously treated with any of the agents tested in the study.

Key exclusion criteria included prior treatment with EGFR inhibitor or BRAF V600E inhibitor; Known hypersensitivity Although our sample size was small, our multiplex immunofluorescence results demonstrated to any investigational agent; Active or history of autoimmune disease; current immunosuppressive medication. Tumors should be MSS/pMMR, as determined by testing (immunohistochemistry, polymerase chain reaction or next-generation sequencing (NGS)).

### Endpoints

The primary endpoints are to evaluate tolerability and safety of the VCC regimen. Secondary endpoints include objective response rate (ORR), disease control rate (DCR), PFS, and OS.

Safety and tolerability will be assessed by monitoring the incidence and severity of AEs and laboratory abnormalities. DLTs were defined as the following events occurring during the AEs evaluation period and judged by the investigator to have a high probability of being related to the investigational drug: Any grade ≥ 3 non-hematological toxicity with the exception of alopecia, grade 3 fever and rash that can be rapidly alleviated after study drug withdrawal and symptomatic treatment can also be excluded; grade 3 thrombocytopenia lasting ≥ 4 days; grade 4 neutropenia lasting ≥ 4 days; grade ≥ 2 immunotherapy-associated cardiotoxicity. ORR is defined as the proportion of patients with the best overall response of the complete response (CR) or partial response (PR) according to RECIST 1.1. DCR is defined as the proportion of patients with the best overall response of CR, PR, or stable disease (SD). OS is defined as the time from randomization to the date of death from any cause. PFS is defined as the time from enrollment to the first documentation of objective disease progression or death from any cause.

### Treatment plan

A course of VCC regimen consists of an intravenous injection of cetuximab 500 mg/m2 and fixed-dose camrelizumab 200 mg repeated every 2 weeks and oral vemurafenib. The administration of vemurafenib was divided into dose level 1 (960 mg once daily) and dose level 2 (960 mg twice daily). Rationale for dose selection: in the SWOG S1406 study, the VIC regimen was administered at the following doses: vemurafenib 960 mg once daily, cetuximab 500 mg/m², and irinotecan 180 mg/m² every two weeks (q2w) [10]. For metastatic malignant melanoma and colorectal cancer with BRAF V600E mutation, the standard dose of vemurafenib is 960 mg twice daily [8, 22, 23]. Treatment was continued until disease progression, intolerable adverse events (AEs), or patient withdrawal. Treatment was discontinued if more than two consecutive doses of vemurafenib were missed due to toxicity, unless the patient was judged to be experiencing clinical benefit.

The dose-limiting toxicity (DLT) observation period began at the first treatment and lasted for 2 weeks. Escalation to the next dose group was permitted only after all participants in the current dose group had completed enrollment and no DLTs were observed within 2 weeks after administration. If 1 out of 3 participants in a dose group 1 experienced a DLT within 2 weeks, the cohort was expanded to include 3 additional participants. If 1 or more of the additional 3 participants experienced a DLT, this dose 1 was defined as the maximum tolerated dose (MTD), and enrollment was halted. If no DLTs occurred in the additional 3 participants, dose escalation proceeded.

The rationale for selecting camrelizumab is as follows: The most common adverse reaction of camrelizumab is reactive capillary endothelial proliferation [24, 25], which is well-recognized by clinicians, and its management strategies are relatively mature and straightforward. In the context of combination therapy with multiple agents, selecting an immunotherapy drug with a well-established and easily manageable safety profile is crucial. Additionally, the relatively lower cost of this drug reduces the financial burden on patients.

### Safety and response assessments

Regular safety assessments were performed based on physical examination, vital signs, ECOG performance status, laboratory parameters, and cardiac assessments. AEs, defined by the National Cancer Institute Common Terminology Criteria for Adverse Events version 5.0, were assessed at each cycle. The relation between AEs and the study drug was assessed by the investigator. The type and incidence of AEs, DLTs, and laboratory examination results were recorded.

Efficacy was evaluated by local investigator assessment using RECIST v1.1 [26]. Tumor assessments were performed at baseline by contrast-enhanced computed tomography with intravenous contrast of the chest and abdomen. Subsequent assessments were performed every 8weeks (+/-1 week) until disease progression or patient withdrawal. Treatment response was assessed as CR, PR, SD, progressive disease (PD), or not evaluated. All patients were observed for survival.

### Immunofluorescence staining

Pre-treatment biopsies were not mandatory. We collected specimens from enrolled patients who had sufficient sample availability. Formalin-fixed, paraffin-embedded, and tumor-containing archival tissue sections obtained at baseline diagnosis were obtained from the pathology department. Immunofluorescence staining was performed to detect the differences in the immune microenvironment in patients with different responses to VCC regimens. Slides were stained using a 5-color, 4-plex mIF assay (CD4, CD8, CD69, forkhead box P3 (FoxP3), and DAPI for nuclei), (CD16, CD56, HLADR, CD11c, and DAPI for nuclei), (programmed cell death ligand 1 (PD-L1), CTLA-4, CD31, CD44, and DAPI for nuclei), (FAP, FSP, α-SMA, Ki-67, and DAPI for nuclei). Slides were imaged on the Tissue Slice Digital Scanner (3DHISTECH, Pannoramic MIDI) to acquire the chromogenic stain image. For each available stained section scan image, the number of cells positive for the above markers was counted using ImageJ software.

### Statistical design

Data from the study were summarized with respect to demographic and baseline characteristics, safety observations and measurements, efficacy observations and measurements, and all relevant positive cell counts using descriptive statistics (mean, standard deviation, median, minimum, and maximum), and contingency tables (frequencies and percentages). Overall response rate (ORR; CR or PR) and disease control rate (DCR; PR, CR, or SD) are summarized with associated 95% confidence intervals (CI) according to RECIST 1.1. Kaplan-Meier method was used to evaluate OS and PFS using SPSS software. Univariate and multivariate analyses of PFS were performed using the COX proportional hazards regression model to determine the association between baseline laboratory test results and secondary parameter results with VCC regimen. Immunofluorescence staining data were analyzed using Graphpad. Prism software version 9.5. Continuous variables are presented as mean ± standard deviation. The independent samples t-tests were applied to compare continuous variables between groups. Statistical significance was set at *p* < 0.05.

## Results

### Patient population

The main process of the study is summarized in Supplemental Figure 1. A total of twelve patients were enrolled in this phase 1 dose-escalation study. One patient experienced a DLT after 6 patients were enrolled in dose level 1, thus dose level 2 was not explored. Another 6 patients were enrolled to explore the safety and tolerability of dose level 1. All patients received at least 1 cycle of VCC regimen at dose level 1.

Baseline patient characteristics are summarized in Table 1. All patients were Asian race, six were female and six were male with a median age of 51.0 years (range 33–75 years). The primary tumor mainly was in the right colon (66.7%). 11 patients (91.7%) had synchronous metastasis, and 9 patients (66.7%) had multiple organ metastasis. The most common metastatic sites were liver (75.0%), distant lymph nodes (50.0%) and peritoneum (50.0%). Histologically, the tumor of 25% patients was mucinous adenocarcinoma phenotype, and the pathology was mainly poorly differentiated (50.0%). All patients were associated with RAS gene wild type, BRAF V600E mutation and MSS status according to NGS. Prior to enrollment, all patients were treated with systemic therapy.

**Table 1:** Baseline characteristics of enrolled patients

Characteristics	Number	Percent (%)
**Age (mean)**	51 years old(33–75)	
**Sex**		
Male	6	50.0%
Female	6	50.0%
**ECOG-PS score**		
0	7	58.3%
1	5	41.7%
**Primary site**		
Right colon	8	66.7%
Left colon	2	16.7%
Rectum	2	16.7%
**Metastatic sites**		
1	3	25.0%
2	2	16.7%
≥ 3	7	58.3%
**Metastatic pattern**		
Synchronous	11	91.7%
Metachronous **Metastasis organ**	1	8.3%
Liver metastasis Peritoneum metastasis Distant-lymph-node Lung metastasis	9 6 6 0	75.0% 50.0% 50.0% 0.0%
**RAS wild-type**	12	100.0%
**pMMR**	12	100.0%
**Differentiation**		
G1-2	3	25.0%
G3	6	50.0%
Unknown	3	25.0%
**Mucinous adenocarcinoma**	3	25.0%
**CD4 + T cell**		
Normal	6	50.0%
Abnormal	4	33.3%
Unknown	2	16.7%
**CD8 + T cell**		
Normal	7	58.3%
Abnormal	3	25.0%
Unknown	2	16.7%
**CD4/CD8**		
Normal	5	41.7%
Abnormal	5	41.7%
Unknown	2	16.7%
**LDH level**		
Normal	8	66.7%
Abnormal	3	25.0%
Unknown	1	8.3%
**NLR (mean)**	2.21(0.90–7.50)	
**PLR (mean)**	165.54(85.80-304.35)	
**Pror surgery treatment**		
No	8	66.7%
Yes	4	33.3%
**Prior lines systemic therapy**		
1	9	75.0%
2	2	16.7%
3	1	8.3%

Abbreviations: G1 = Well differentiated; G2 = Moderately differentiated; G3 = Poorly differentiated; ECOG-PS: Eastern Cooperative Oncology Group performance status scale; pMMR: perfect mismatch repair; NLR: neutrophil to lymphocyte ratio; PLR: plastocyte to lymphocyte ratio

### Safety

According to NCI-CTCAE v5.0 evaluation criteria. Grade 1–2 adverse reactions occurred in all 12 patients. The most common grade 1–2 AEs were drug-related rash (75.0%), joint pain (75.0%), reactive capillary hyperplasia (66.7%), anemia (53.3%), drug-induced fever (41.7%), muscle pain (41.7%), and mucosal bleeding (41.7%). 6 patients (50.0%) underwent grade 3 treatment-related adverse reactions, including fever (25%), drug-related rash (16.7%), mucosal bleeding (8.3%), immune myocarditis (8.3%), anemia (8.3%), and thrombocytopenia (8.3%) (Table 2). One patient developed grade 3 drug-induced rash, and the rash was relieved after stopping the treatment of vemurafenib and using loratadine anti-allergic treatment for 5 days (Supplement Figure 2).

**Table 2:** Adverse events of enrolled patients treated with VCC regimen

Adverse events	All grade	Grade 1–2	Grade 3	Grade 4
	12(100%)	12(100%)	6(50.0%)	0
**Hematological toxicity**				
Neutrophil count decreased	1 (8.3%)	1 (8.3%)	0	0
Anemia	8(66.7%)	7(53.3%)	1 (8.3%)	0
Thrombocytopenia	2 (16.7%)	1 (8.3%)	1 (8.3%)	0
ALT、AST increased	1 (8.3%)	1 (8.3%)	0 (0%)	0
**Gastrointestinal**				
Diarrhea	1 (8.3%)	1 (8.3%)	0	0
**Constitutional**				
Fatigue	3 (25.0%)	3 (25.0%)	0	0
**Joints and muscles**				
Joint pain	9(75.0%)	9 (75.0%)	0	0
Muscular pain	5 (41.7%)	5 (41.7%)	0	0
**Skin and mucous membranes**				
Rash	11(91.7%)	9 (75.0%)	2 (16.7%)	0
Mouth ulcers	2 (16.7%)	2 (16.7%)	0	0
Oral hemangiomas	2 (16.7%)	2 (16.7%)	0	0
Mucosal bleeding	6 (50.0%)	5 (41.7%)	1 (8.3%)	0
Reactive capillary proliferation	8(66.7%)	8(66.7%)	0	0
**Immune system**				
Immune myocarditis	1 (8.3%)	0	1 (8.3%)	0
Immune thyrotoxicosis	1 (8.3%)	1 (8.3%)	0	0
**Other**				
Fever	8(66.7%)	5 (41.7%)	3 (25.0%)	0

**Fig. 1:**
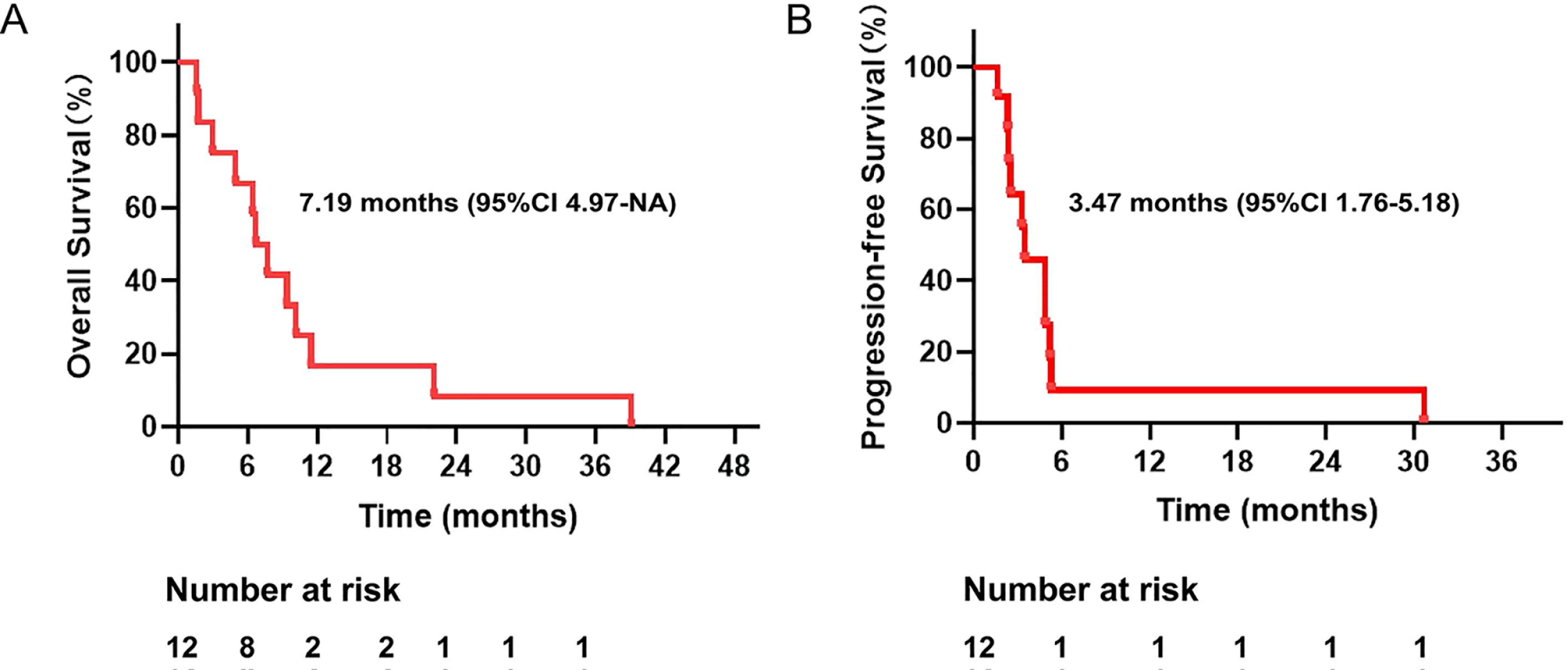
(**A**): Kaplan-Meier curves of OS in BRAF V600E mutant mCRC patients treated with VCC regimen; (**B**): Kaplan-Meier curves of PFS in BRAF V600E mutant mCRC patients treated with VCC regimen. Abbreviations: DLT: dose-limiting toxicity; MSS: microsatellite stable

**Fig. 2:**
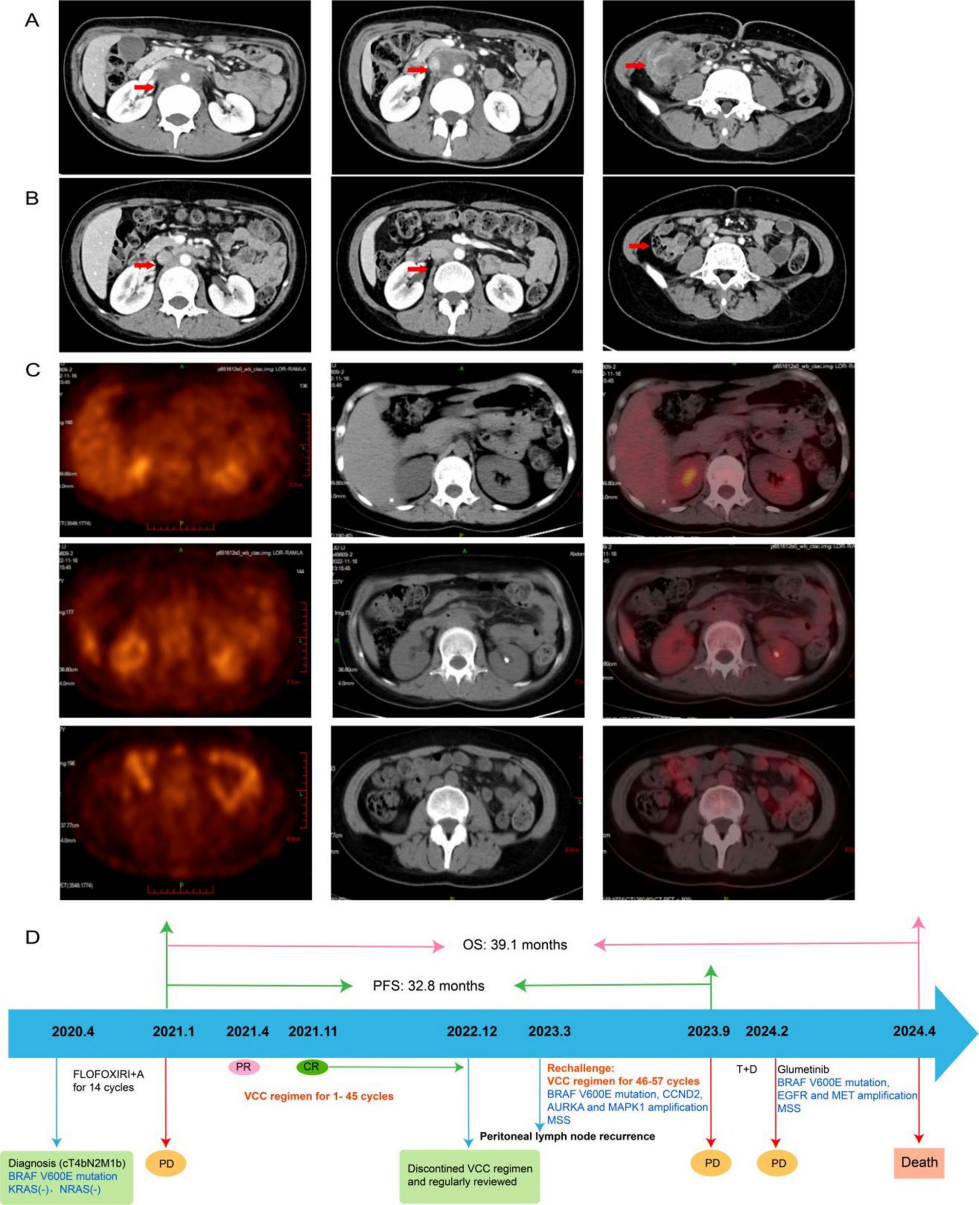
(**A**): Baseline CT images of a patient showed tumors in both intestinal and retroperitoneal lymph nodes. (**B**): The tumors in both intestinal and retroperitoneal lymph nodes were complete regressed after receiving 30 cycles VCC regimen. (**C**): PET-CT images of the patient achieving complete remission. Abbreviations: OS: overall survival; PFS: progression-free survival; CR: complete response; PR: partial response; PD: progressive disease; A: bevacizumab; 2.T + D: trametinib + dabrafenib’ TMB: tumor mutation burden; MSS: microsatellite stable

A total of 2 patients underwent DLT: One patient had grade 3 thrombocytopenia and grade 3 immune myocarditis and discontinued the treatment, which required hospitalization for the administration of methylprednisolone and gamma globulin shock but resolved without sequelae. One patient had grade 3 vaginal bleeding. The patient treated with endometrial curettage surgery and no vaginal bleeding occurred after surgery. One patient died of electrolyte disturbance from intestinal obstruction during the medication interval because of disease progression.

### Efficacy

**Fig. 3:**
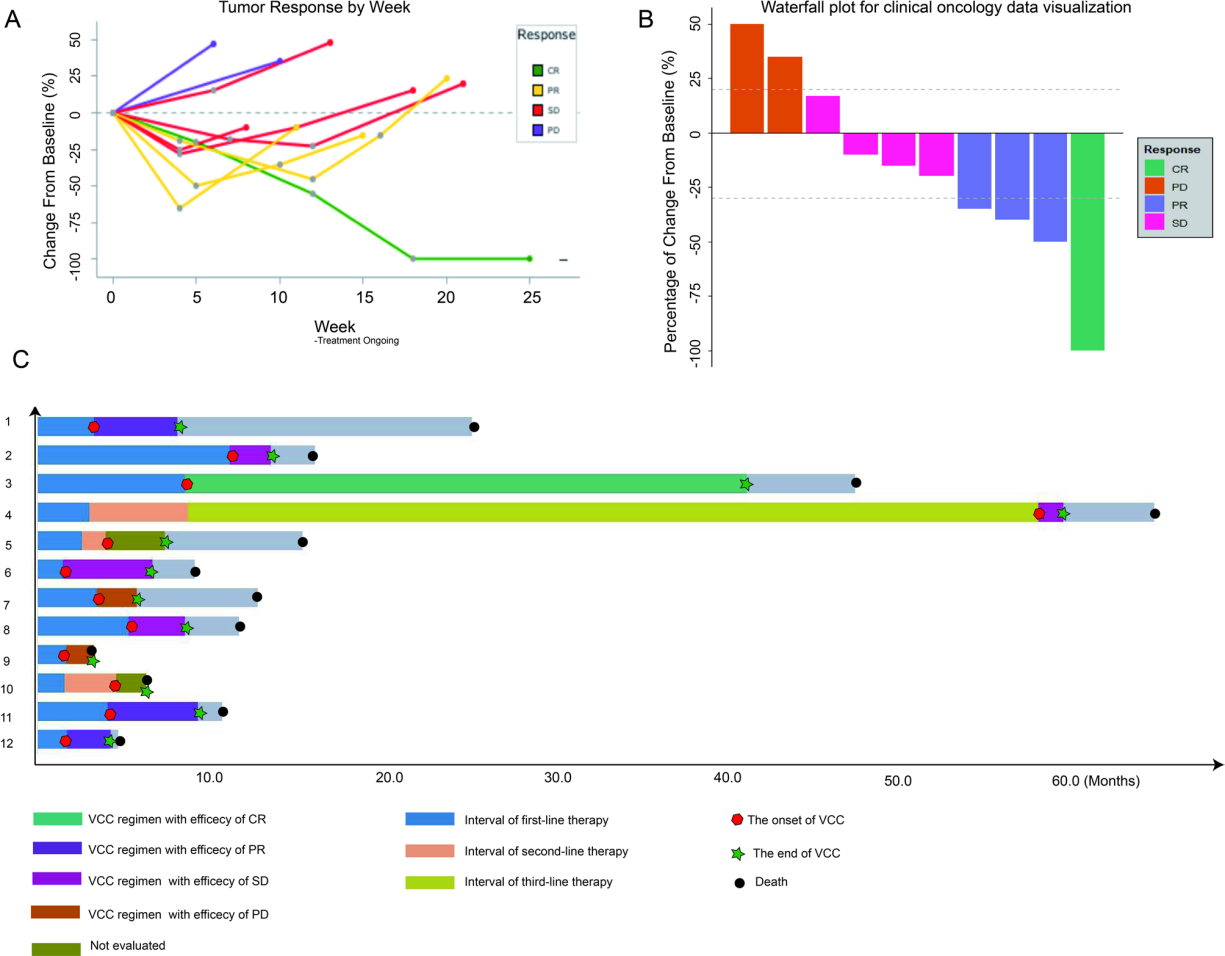
Spider plot, waterfall plot and swimmer plot of BRAF V600E mutant mCRC patients treated with VCC regimen. (**A**) Spider plot; (**B**) waterfall plot; (**C**) swimmer plot. Abbreviations: CR: complete response; PR: partial response; SD: stable disease; PD: progressive disease

Among 12 patients, 1 patient stopped the trial because of grade 3 immune myocarditis after using 1 cycle VCC regimen, 1 patient died of electrolyte disturbance from intestinal obstruction, thus 10 patients received efficacy evaluation. According to the RECIST 1.1 evaluation criteria, 1 patient (8.3%) achieved CR, 3 patients (25.0%) achieved PR, 4 patients (33.3%) achieved SD, and 2 patients (16.7%) achieved PD. The ORR was 33.3%, and DCR was 66.7%. The median OS of 12 patients was 7.19 months (95% CI 4.97-NA), and the median PFS was 3.47 months (95% CI 1.76–5.18 months) (Fig. 1 and B). One patient achieved continuous CR after 30 cycles of treatment, and the trial protocol has been discontinued. The continuous remission time has been as long as 32.8 months and the OS achieved 39.1 months (Fig. 2). The spider diagram and waterfall diagram were made according to the time of medication, the best efficacy during the period, the tumor regression and progression (Fig. 3).

The standard range of CD4 + T cells count is 471–1220 cell/ul. The standard range of CD8 + T cells count is 303–1003 cell/ul. The standard range of CD4/CD8 ratio is 0.97–2.31.

We conducted an exploratory analysis to explore the association between baseline laboratory tests (the number of CD4 + T cells, the number of CD8 + T cells, CD4/CD8 ratio) with secondary parameters (NLR, PLR) and PFS of BRAF V600E mutated mCRC patients treated with VCC regimen. We performed univariate and multivariate analysis of PFS of VCC regimen and found that abnormal baseline CD4/CD8 ratio (< 0.97 or > 2.31) was associated with poor PFS (Table 3). The sample size is small, so this hypothesis results require validation in a larger phase II study.

### Immune environment

**Fig. 4:**
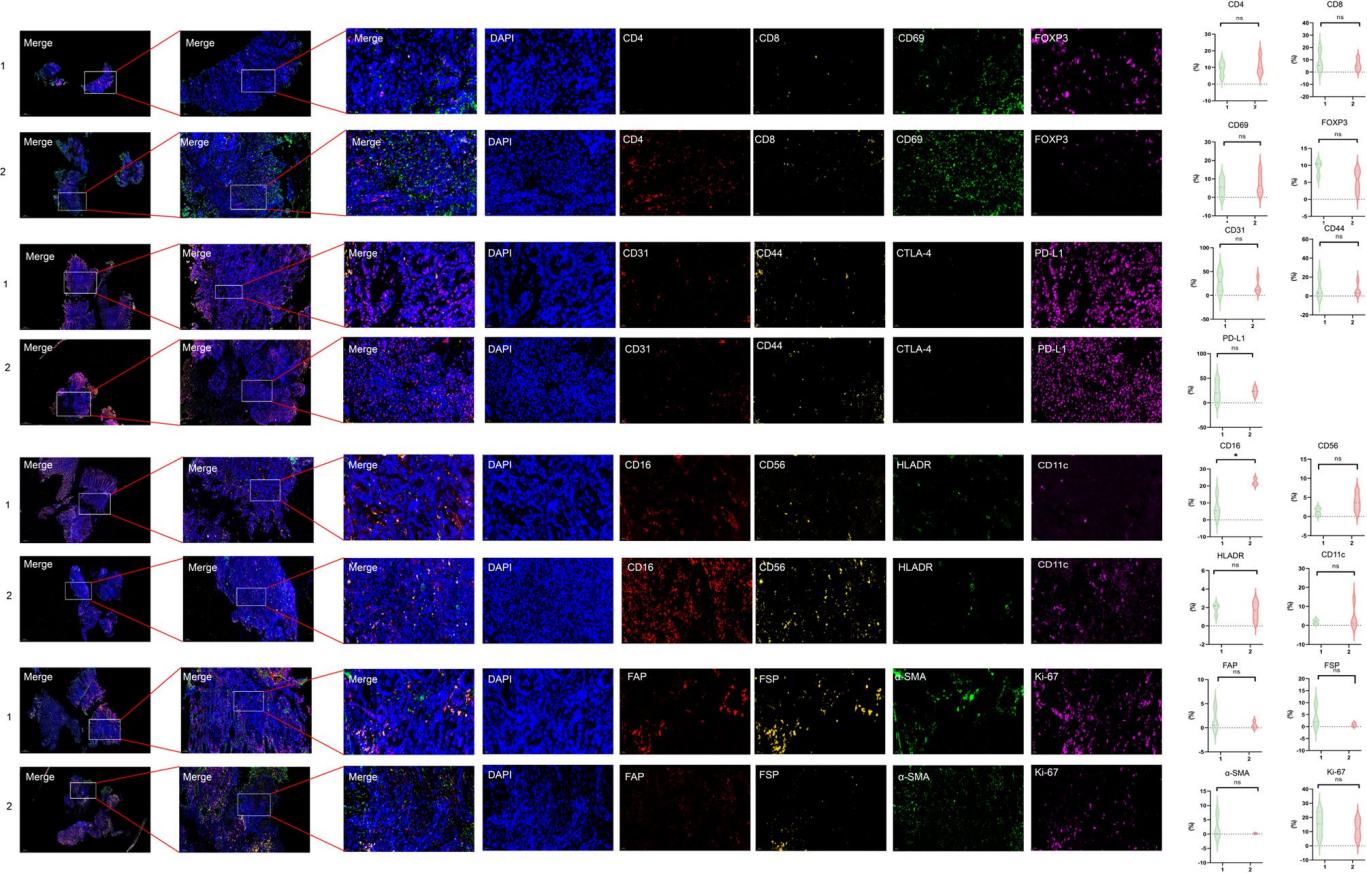
Comparison of immunofluorescence staining between two groups with different efficacy for VCC regimens (group 1 includes patients that did not achieve DCR, group 2 includes patients that achieved DCR)

We extracted the seven patients ‘s pathological sections obtained at baseline diagnosis. pre-treatment biopsies were not mandatory. The specimen was derived from the puncture or surgery of the primary or metastatic site. According to the efficacy to VCC regimen, those patients were divided into group 1(response of PD) and group 2 (response of CR + PR + SD), with 3 patients in group 1 and 4 patients in group 2. The results of Immunofluorescence staining discovered that the group 2 with better response is associated with higher levels of CD4(mean:11.080 vs 9.137, *p* = 0.627), CD69 (mean: 7.562 vs. 5.383, *p* = 0.626)and lower levels of CD8 (mean: 5.929 vs 8.929, *p* = 0.568), Foxp3 (mean: 5.567 vs. 9.263, *p* = 0.150), CD31 (mean: 18.040 vs. 28.520, *p* = 0.537), and CD44 (mean:6.710 vs. 8.065, *p* = 851) compared with group 1. The number of NK cells is increased in the group 2 [CD16 (mean:22.300 vs 6.982, *p* = 0.011) and CD56(mean: 3.799 vs. 1.799, *p* = 0.243). The number of DC is also increased in group 2 (CD11c) (mean: 4.277 vs 2.017, *p* = 0.656). Furthermore, the tumor with better efficacy is infiltrated with less fibroblasts, including α-SMA (mean: 0.174 vs 2.412, *p* = 0.314), FAP (mean: 0.546 vs. 1.677, *p* = 0.393), FSP (mean:0.695 vs. 4.078, *p* = 0.201). The expression of Ki-67 is decreased in group 2 (mean: 11.910 vs 15.320, *p* = 0.586). (Fig. 4). The immunofluorescence data are lack of statistical significance due to the small sample size. This hypothesis results require validation in a larger phase II study.

## Discussion

The results of this phase I study indicate that vemurafenib (at doses of 960 mg daily), cetuximab with camrelizumab (VCC) regimen was moderately tolerated and associated with a preliminary efficacy in previously treated mCRC patients with BRAF V600E mutation, with an ORR of 33.3%.

There are challenges encountered in patient recruitment or follow-up. BRAF V600E mutations account for only approximately 5–15% of mCRC cases and eligible patients must have no prior exposure to BRAF/EGFR inhibitors and be MSS-type, limiting the number of eligible candidates, which requiring single-center studies to screen large patient populations over extended periods to meet sample size requirements. Some patients are excluded due to organ dysfunction, poor ECOG performance status, or comorbidities. Some eligible patients decline participation due to transportation barriers, treatment costs, or preference for standard therapy. During follow-up, patients may seek care at other medical institutions, complicating data collection. When patients are lost to follow-up, telephone-based tracking becomes necessary, potentially reducing data reliability. Last, the single-center study population may reflect regional characteristics or treatment preferences of the center, limiting the generalizability of results.

In this study, grade 1–2 AEs mainly included drug-induced rash (75.0%), joint pain (75.0%), reactive capillary endothelial proliferation (66.7%), anemia (53.3%), drug-induced fever (41.7%), muscle pain (41.7%), mucosal bleeding (41.7%). Joint muscle pain, fever, and mucosal bleeding were common adverse reactions of vemurafenib. Reactive capillary hyperplasia is a side effect of carrelizumab, which can damage capillaries in the skin, thereby stimulating local hyperplasia to form red rash. The incidence of grade 3 and above AEs in this study was 50.0%, which was consistent with the incidence of encorafenib + cetuximab double-target regimen (50.0%) and encorafenib + binimetinib + cetuximab triplet-therapy group (58.0%) in BEACON study [27], suggesting that double-target regimen combined with immunotherapy did not significantly increase the incidence of AEs. The incidence of grade 3 and above AEs in this study was lower than that in the IMPROVEMENT study (71.4%) [28]. IMPROVEMENT study explored the efficacy of cetuximab with vemurafenib plus FOLFIRI regimen in BRAF V600E mutant mCRC. The high incidence in that study may be attributed to the high dose of vemurafenib (960 mg twice a day) and the high incidence of grade 3 neutropenia caused by FOLFIRI (38.1%). In this study, grade 3 drug-related rash (16.7%), which was similar to that of our study [28]. Treatment was discontinued in one case with grade 3 immune myocarditis, while the remaining patients did not undergo dose reduction or treatment interruption. In our study, the high incidence of grade 3 fever in our study was considered to be caused by vemurafenib. Fever usually occurred after the treatment of a cycle VCC regimen. All the patients recovered after symptomatic cooling, and no fever occurred in the subsequent use of VCC regimen. In conclusion, this VCC regimen is tolerated.

Notably, one patient underwent immune myocarditis and treated with urgent intravenous glucocorticoids therapy (2 mg/kg daily) supplemented with oxygen inhalation and diuretic administration for symptomatic relief. Following three days of corticosteroid therapy, the patient’s symptoms including fatigue, chest tightness, and dyspnea showed significant improvement, accompanied by a marked reduction in cardiac biomarkers upon re-examination. Following gradual tapering of glucocorticoids, the patient’s related symptoms resolved completely, and cardiac biomarkers returned to normal ranges without long-term sequelae. Immunotherapy was not reintroduced in this patient thereafter. According to previous studies, camrelizumab-related adverse events primarily manifest as reactive capillary endothelial proliferation, hypothyroidism, hyperthyroidism, alanine aminotransferase increased, and immune-mediated enterocolitis [29, 30]. Camrelizumab-related myocarditis has been previously reported in the literatures [31, 32]. Mechanically, pyroptosis-mediated by GSDME in cardiomyocytes facilitates ICIs-associated myocarditis via mediating mitochondrial damage and releasing mitochondrial DNA (mtDNA), further activating cyclic GMP-AMP synthase (cGAS)-stimulator of interferon genes (STING) signaling pathway [33]. Cardiac-myosin-specific autoreactive T cells is also an important mediator in ICIs-associated myocarditis [34]. The following strategies should be implemented to prevent and manage immune myocarditis in patients receiving immunotherapy. Before treatment, for patients undergoing immunotherapy, particularly those with high-risk factors such as pre-existing autoimmune diseases, cardiovascular disorders, or prior exposure to cardiotoxic agents, a comprehensive cardiac evaluation is required before treatment initiation. During immunotherapy, regular monitoring of ECG, cardiac enzymes (e.g., creatine kinase, troponin), and echocardiography is essential. Intensive monitoring is particularly critical during the first 6 weeks of treatment. Clinicians should remain vigilant for non-specific symptoms such as chest pain, dyspnea, palpitations, or fatigue, and promptly investigate potential myocarditis [35, 36].

The following measures are beneficial for reducing the adverse reactions observed in this study: 1. Optimize the dosing regimen by replacing the mode of administering all drugs at full dose simultaneously. A stepwise dosing approach can be adopted, where BRAF inhibitors and EGFR inhibitors are used alone first, and after the peak of acute toxicities has passed (e.g., after 1–2 weeks), the PD-1 inhibitor (camrelizumab) can be introduced.2. Establish a standardized prophylactic medication protocol, such as the preemptive use of topical corticosteroid creams to prevent and treat skin rash commonly associated with BRAF inhibitors. 3. Implement patient selection and risk stratification by excluding patients with pre-existing autoimmune diseases or clinically significant risk factors for myocarditis or pneumonitis (e.g., abnormal cardiac markers, interstitial lung disease). 4. Explore predictive biomarkers by focusing on identifying biomarkers associated with severe toxicities. 5. Enhance monitoring and proactive management during treatment.

In the prospective phase I study, the median OS of the enrolled patients was 7.19 months, the median PFS was 3.47 months. And the ORR was 33.3%, the DCR was 66.7%. The ORR of dual-target regimen (encofenib plus cetuximab) was 19.5% in BEACON study [27] and 17.0% of VIC regimen (vemurafenib + cetuximab + irinotecan) in SWOG S1406 study [10], which indicated that dual-target regimen plus immunotherapy may further improve the efficacy. The median PFS in our study was shorter than that in doublet-therapy group and in triplet-therapy group of BEACON study (3.47 vs. 4.2 vs. 4.3months). The median OS in our study was also shorter than that in doublet-therapy group and in triplet-therapy group (7.19 vs. 8.4 vs. 9.0months). A possible explanation is that our study included a higher proportion of patients with extensive metastatic disease (≥ 3 metastasis sites: 58.3% vs. 53% vs. 51%), as well as a greater rate of liver metastases (75.0% vs. 39% vs. 36%) compared to BEACON study [27]. Our previous retrospective observational study revealed that BRAF V600E mutated colorectal cancer patients with and liver metastases had poorer prognosis and inferior treatment response [37]. Other research centers have also explored the efficacy of targeted therapy combined with immunotherapy in patients with BRAF V600E mutation mCRC. In 2022, ASCO published a phase I/II study to explore the efficacy of Encorafenib combined with Cetuximab and Nivolumab in the treatment of MSS/BRAF V600E mutant mCRC patients. The results showed that the ORR was 50%, the DCR was 96%, and the median PFS and OS were 7.4 months and 15.1 months, respectively. Furthermore, in 2023, the Cancer Center of Massachusetts Hospital [38] found that dabrafenib combined with trametinib plus PD-1 monoclonal antibody inhibitor (sparatlizumab) significantly enhanced the response rate to 24.3% in BRAF V600E mutant mCRC patients. Compared with BRAF inhibitor combined with MEK inhibitor, triplet regimen improved the PFS (4.3 vs. 3.5 months). The median OS achieved 13.6months. The difference in efficacy between VCC regimen and this study regimen may be attributed to the fact that our study enrolled a higher proportion of patients who had received multiple prior lines of therapy (100.0% vs. 89.2%) [38]. Additionally, the inhibitory capacity of EGFR inhibitors and MEK inhibitors against MAPK pathway feedback activation may differ. Furthermore, distinct targeted agents exhibit varying effects on remodeling the immune microenvironment. The use of different PD-1 monoclonal antibodies in the two studies may also have contributed to the efficacy disparities. Future larger-scale studies are needed to directly compare the efficacy of BRAF V600E inhibitor combined with EGFR inhibitor and PD-1 monoclonal antibody versus BRAF V600E inhibitor combined with MEK inhibitor and PD-1 monoclonal antibody.

The efficacy difference of VCC regimen may be attributed to the difference of tumor immune environment. We found that abnormal baseline CD4/CD8 ratio was associated with poor PFS. Previous reports have shown that the ratio of CD4/CD8 T cells reflects the immune system status as a surrogate marker of immune-senescence, and may independently predict all-cause mortality [39, 40]. The decreased CD4/CD8 ratio was significantly associated with the poorer prognosis of patients with cervical carcinoma [41] and patients with nasopharyngeal carcinoma [42].

The patients with better response are associated with higher levels of CD4 + T cells and lower number of Treg cells. Furthermore, CD31and CD44 is also overexpressed in patients with better response. CD31 is a cell surface protein that is expressed on vascular endothelial cells. The expression of CD31 can be used to evaluate the tumor angiogenesis activity in colorectal cancer. The strong positive expression of CD31 suggests that tumor cells have the characteristics of vascular endothelial differentiation, which may be related to tumor growth and metastasis [43]. CD44 has the function of maintaining the activity of colorectal cancer stem cells. High expression of CD44 is associated with poor prognosis [44]. The number of NK cells and DC are also decreased in patients with worser efficacy, while the fibroblasts are increased. Increased fibroblasts can secrete VEGF to increase the rate of angiogenesis, and participate in the immune escape of cancer cells [45]. Except for NK cells, the differences of other cells were not statistically significant, which may be contributed to the small sample in our study. More studies with larger sample size are needed to verify this conclusion in the future.

Although our sample size was small, our multiplex immunofluorescence results demonstrated a correlation between the tumor microenvironment and treatment efficacy. Due to the invasive nature of biopsy procedures, we were unable to obtain post-treatment tumor specimens from patients, which prevented comparative analysis of immune microenvironment changes following VCC regimen treatment. According to previous studies, while targeted combination therapy alone provides limited clinical benefit, the addition of immunotherapy enhances treatment efficacy. The potential mechanism may involve BRAF pathway inhibitors downregulating MAPK signaling, thereby reversing the immunosuppressive properties of tumor cells (e.g., by increasing CD8⁺ T cell infiltration) and simultaneously enhancing tumor cell-intrinsic immune-related expression. This process converts “cold tumors” into “hot tumors,” thereby establishing a foundation for synergizing with PD-1 inhibitor therapy [14, 38, 46].

Among our enrolled patients, one patient achieved long-term survival with complete response. This patient received the experimental regimen as second-line therapy. The prior first-line treatment consisted of 12 cycles of FOLFOXIRI plus bevacizumab, resulting in a median PFS of 8.57 months. The relatively prolonged PFS during first-line therapy and second-line therapy suggests unique biological characteristics of this patient’s tumor. Future phase II studies should include broader populations to investigate whether patients demonstrating superior treatment response possess distinct biological features. In the future, the safety and efficacy of different targeted immunotherapy combination strategies for BRAF V600E-mutant colorectal cancer can be further explored, such as combining BRAF V600E inhibitors with MEK inhibitors and PD-1 monoclonal antibodies. Additionally, it is necessary to further identify biomarkers associated with efficacy in the benefiting population to facilitate personalized precision treatment for patients.

### Limitations

In this prospective study, we only enrolled patients from a single center, which may introduce patient selection bias. Additionally, with only 12 patients included, the sample size remains limited. We analyzed the Immunofluorescence staining in the immune microenvironment in only seven patients. The biomarker analysis should be considered exploratory, and these findings require validation in larger Phase II studies.

## Conclusions

VCC regimen exhibited manageable adverse reactions and efficacy in BRAF V600E-mutated/MSS patients with metastatic colorectal cancer who progressed after standard treatment. The different antitumor activity may be explained by different immune environment of tumors. This is a pilot study and a larger, randomized phase II trials is planned to validate the findings.

**Table 3:** Univariate and multivariate analysis of PFS of VCC regimen for enrolled patients

Variates	Univariate			Multivariate		
	HR	95.0% CI	*p*	HR	95.0% CI	*p*
**NLR**						
≤ 3.07	Reference					
>3.07	1.668	0.435–6.395	0.445	2.474	0.089–69.053	0.594
**PLR**						
≤ 144	Reference			1		
>144	2.170	0.529–8.899	0.282	2.054	0.127–33.316	0.613
**CD4 + T cell**						
471–1220 cell/ul	Reference					
< 471 or > 1220cell/ul	1.794	0.477–6.473	0.387	0.595	0.1-3.531	0.568
**CD8 + T cell**						
303–1003 cell/ul	Reference					
< 303 or > 1003 cell/ul	1.369	0.326–5.759	0.668	0.617	0.062–6.171	0.681
**CD4/CD8**						
0.97–2.31	Reference					
< 0.97 or > 2.31	6.371	1.173–34.605	**0.032**	15.848	1.439-174.565	**0.024**

Abbreviations: NLR: neutrophil to lymphocyte ratio; PLR: plastocyte to lymphocyte ratio

## Electronic supplementary material

Below is the link to the electronic supplementary material.

Supplementary Material 1

Supplementary Material 2

## Data Availability

If you need the data, please contact the corresponding author (qiumeng@wchscu.cn).
