# Supplementary figures and images for: Vemurafenib, cetuximab and camrelizumab in BRAF V600E-mutated/MSS metastatic colorectal cancer
Source: J Transl Med. 2025 Nov 12;23:1274. doi: 10.1186/s12967-025-07312-6 (PMC12613881; doi:10.1186/s12967-025-07312-6)

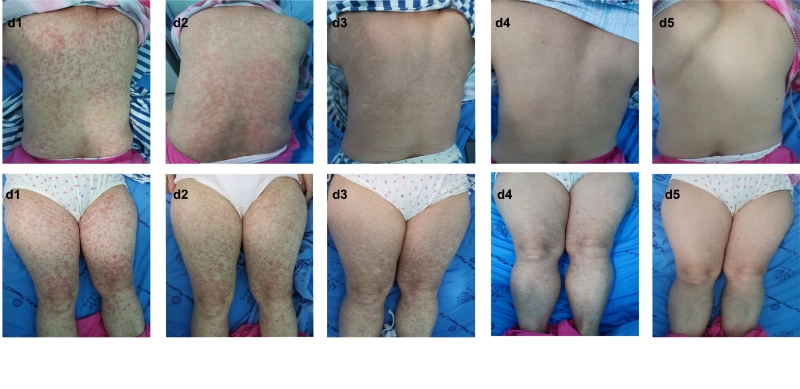

Supplement: Supplementary file 1 — Supplementary Material 1 [file 12967_2025_7312_MOESM1_ESM.jpg]

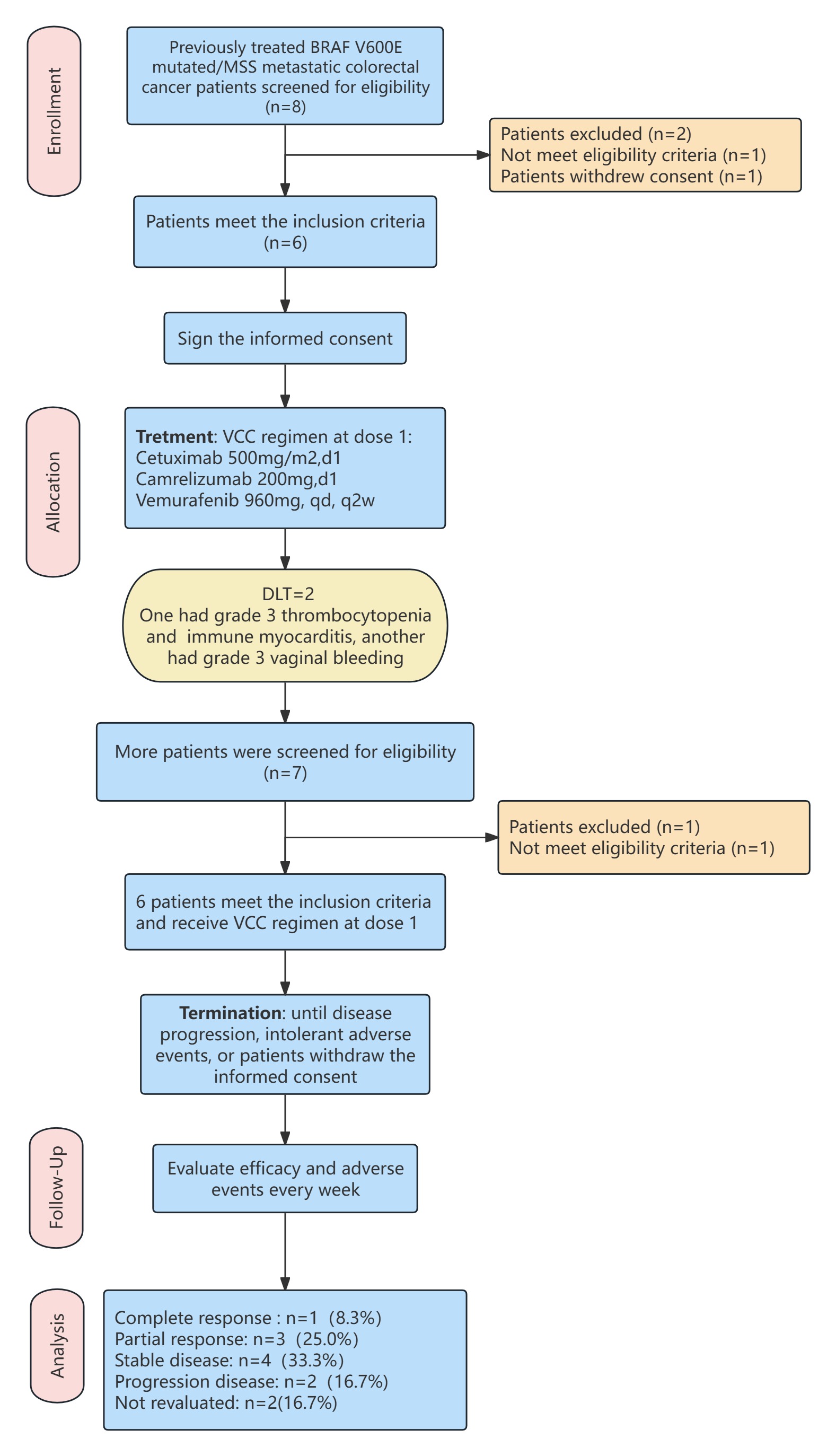

Supplement: Supplementary file 2 — Supplementary Material 2 [file 12967_2025_7312_MOESM2_ESM.jpg]
